# Real-world study on the effectiveness and breast safety analysis of hormone replacement therapy during menopause

**DOI:** 10.3389/fmed.2026.1817851

**Published:** 2026-06-02

**Authors:** Yingxia Wang, Yaqin Liu, Jing Zeng, Mengyi Wang, Yuhuan Li, Yinglan Wu, Jie Gao, Xia Chen

**Affiliations:** 1 Department of Women Health Care, Hunan Provincial Maternal and Child Health Care Hospital, Changsha, Hunan, China; 2 Department of Women Health Care, Huaihua Maternal and Child Health Hospital, Huaihua, Hunan, China

**Keywords:** bone mineral density, breast safety, Kuppermann score, menopausal hormone therapy, postmenopausal women

## Abstract

**Objective:**

This real-world study evaluated the effects of 24-month menopausal hormone therapy (MHT) on bone mineral density (BMD), menopausal symptoms, and breast safety in postmenopausal women.

**Methods:**

A total of 809 postmenopausal patients aged 40–60 years receiving MHT from June 2021 to June 2022 were enrolled. Kuppermann scores, BMD, and breast findings were assessed at baseline and at 6, 12, 18, and 24 months. Changes in outcomes were analyzed across time points, with Bonferroni correction for *post hoc* pairwise comparisons.

**Results:**

Over the 24-month period, the prevalence of osteoporosis and low bone mass decreased significantly from 18.0 to 14.0% and from 27.4 to 21.4%, respectively (*p* < 0.001). Significant differences in BMD were observed between baseline and each post-treatment time point (all *p* < 0.001), and BMD presented staged changes after treatment. The mean Kuppermann score demonstrated marked improvement, declining from 40.17 ± 6.04 at baseline to 11.38 ± 4.90 at 24 months (*p* < 0.001), with the pace of symptom relief exhibiting distinct phases. No significant changes were observed in breast density, volume, or the prevalence of calcifications or masses (all *p* > 0.05).

**Conclusion:**

The 24-month MHT regimen effectively improved BMD, alleviated menopausal symptoms in a stage-dependent manner, and demonstrated a favorable breast safety profile. These findings support the role of MHT in the comprehensive management of postmenopausal health.

## Highlights

- This real-world study followed 809 postmenopausal women over 24 months.
- MHT significantly improved bone mineral density and reduced bone mass loss.
- Menopausal symptom relief showed a distinct, stage-dependent improvement pattern.
- No significant adverse effects were observed on breast density or lesion prevalence.
- Findings support MHT as an effective intervention for postmenopausal health management.

## 1 Introduction

With the acceleration of global population aging, menopausal women’s health management has become a key focus in the field of public health. Importantly, the life expectancy of global women soars more than that of men due to longer telomere and several other protective factors, such as the genetic buffer provided by two X chromosomes and the antioxidant protection of estrogen. According to the World Health Organization’s prediction, by 2030, the global number of perimenopausal and postmenopausal women will reach 1.2 billion, among which the number of menopausal women in China has risen to the top in the world, exceeding 200 million, and continues to grow (1). The decline in estrogen levels due to ovarian function decline after menopause can lead to a series of physiological and psychological abnormalities. Approximately 50–60% of women experience vasomotor symptoms such as hot flashes and night sweats, with 10–15% reporting severe symptoms that significantly impair quality of life (2). Concurrently, menopause increases the long-term risk of chronic diseases, including osteoporosis and cardiovascular disease (3, 4). These symptoms not only interfere with sleep, mood, and sexual function (5), but may also limit the mobility of working women, reduce work efficiency, and even force them to adjust their career plans. Furthermore, the neglected peri-menopausal symptoms could affect Gross Domestic Production (GDP) negatively, though nobody has surveyed it so far. All these consequences result in significant personal health burdens and socio-economic impacts.

Menopause hormone therapy (MHT), as a core intervention for hormone deficiency, has been clinically validated for alleviating menopause-related symptoms, improving urogenital atrophy, and preventing osteoporosis (6–8). Particularly when initiated during early menopause (within 10 years of menopause or before age 60 years old), MHT maximizes benefits while minimizing long-term risks, establishing it as one of the standard protocols recommended by global guidelines (9–11). In recent years, the safety cognition and clinical application norms of MHT have been constantly updated and improved. In 2024, the International Federation of Gynecology and Obstetrics (FIGO) released a position statement focusing on how to interpret the benefits and risks of MHT for menopausal women, and the International Menopause Society (IMS) also issued the 2024 white paper concerning core controversies over menopause and menopausal hormone therapy (12, 13). Meanwhile, the FDA revised relevant drug labeling and canceled the traditional boxed warnings for MHT in 2025, further optimizing the risk–benefit evaluation system for clinical medication (14) However, MHT’s clinical application remains constrained by safety concerns, including controversies over potential risks such as breast cancer, endometrial cancer, and thromboembolism, coupled with public misconceptions about hormone therapy. These factors have resulted in persistently low rates of standardized menopausal treatment in China.

Based on the above background, this study conducted a 2-year real-world clinical follow-up of postmenopausal women in China to systematically evaluate the effects of menopausal hormone therapy (MHT) on vasomotor symptoms and quality of life, while monitoring treatment-related adverse events. The aim is to provide high-quality evidence for clarifying the true benefit–risk profile of MHT in the Chinese population, and to offer data support for the standardized clinical application of MHT and the improvement of health management for menopausal women.

## 2 Methods

### 2.1 Study population

This study adopted a real-world prospective cohort study design. Postmenopausal women aged 40–60 years who visited the Menopause Clinic of Hunan Provincial Maternal and Child Care Health Hospital from June 2021 to June 2022 were selected as the study subjects. Inclusion criteria: (1) Diagnosis of perimenopausal syndrome according to the modified Kupperman score, meeting the perimenopausal diagnostic criteria of the Stages of Reproductive Aging Workshop (STRAW) criteria (15), (2) received MHT intervention and underwent regular follow-up for at least 2 years. Exclusion criteria: (1) Previous use of MHT or other sex hormones within 12 months, (2)Severe hepatic or renal disease, or uncontrolled thyroid dysfunction, (3) Unexplained vaginal bleeding, (4) History of breast cancer, endometrial cancer, ovarian cancer, or other hormone-related malignancies, (5) Use of anti-osteoporosis medications (bisphosphonates, denosumab, raloxifene, teriparatide) within 12 months, (6) Long-term systemic glucocorticoid use (≥3 consecutive months), (7) Rheumatoid arthritis, ankylosing spondylitis, or other disorders affecting bone metabolism, (8) Type 1 diabetes or uncontrolled type 2 diabetes, (9) Contraindications to MHT. All study subjects signed informed consent forms and voluntarily underwent regular health examinations, bone density scans, breast examinations, and follow-up visits. This study was approved by the Medical Ethics Committee of Hunan Provincial Maternal and Child Health Care Hospital (Ethical Review Approval Number: 2020-S040).

### 2.2 Data collecting

During the period from June 2021 to June 2022, basic information of the research subjects who met the established study criteria was collected from the menopause clinic, including age, place of residence, ethnicity, education level, occupation, and monthly household income.

### 2.3 MHT intervention

Hormone regimens were selected based on patient preferences regarding menstrual bleeding and uterine status: (1) Cyclic sequential therapy: Femoston (17β - estradiol + dydrogesterone), (2) Continuous combined therapy: 17β - estradiol/dydrogesterone, (3) Estrogen - only therapy: oral or transdermal 17β - estradiol (for hysterectomized women). Follow-up assessments were conducted at 6, 12, 18, and 24 months post-initiation, including blood routine tests, liver and renal function tests, glucose and lipid profiles, bone mineral density measurements, and breast evaluations. Venous thromboembolism risk was systematically evaluated before MHT initiation, and no venous thromboembolic events occurred during the entire study period.

### 2.4 Bone density examination

BMD was measured by dual-energy X-ray absorptiometry (DEXA; DPX BRAVO, GE). Measurement sites: lumbar spine L2-4, femoral neck, total hip. Based on the diagnostic criteria recommended by the World Health Organization (WHO), bone mass is evaluated using the T-score, which is calculated as (measured value - peak bone mass) / standard deviation of normal adult bone density. According to the diagnostic criteria, a T-score >−1 indicates normal bone mass; − 2.5 < T-score ≤−1 indicates osteopenia; and T-score ≤−2.5 indicates osteoporosis. Quality control: daily phantom scanning; no machine drift. Short-term *in vivo* precision: lumbar spine CV < 1.0%, femoral neck CV < 1.5%. Least significant change (LSC): lumbar 2.7%, femoral neck 3.8%.

### 2.5 Mammography

A professional radiologist conducts mammography using the Selenia Dimensions full-field digital mammography system (manufactured by Hologic, USA). The system incorporates Computer-Aided Detection (CAD) technology and is equipped with Quantra software, which calculates breast density, breast fibrous tissue volume, and breast volume based on the total breast volume. The system detects breast calcifications and breast masses.

### 2.6 Statistical analysis

Statistical analyses were performed using SPSS software (version 26.0, IBM Corp., Armonk, NY, USA). Categorical data are expressed as frequencies and percentages. For the comparison of binary outcome variables across multiple time points, Cochran’s *Q* test was applied, followed by post-hoc pairwise analysis using the McNemar test with Bonferroni correction. For categorical variables with multiple outcomes across time points, the Friedman’s *M* test was employed, with subsequent pairwise comparisons conducted using the Wilcoxon signed-rank test and Bonferroni adjustment. Continuous data are presented as mean ± standard deviation (mean ± SD). Changes across time points for these variables were assessed using repeated-measures analysis of variance (ANOVA), with Bonferroni-corrected post-hoc tests for pairwise comparisons. A two-sided *p*-value of < 0.05 was considered statistically significant. The sample size was calculated based on the primary outcome (rate of improvement in vasomotor symptoms) using the McNemar’s Test formula. Parameters were set as follows: *α* = 0.05 (two-sided, *Z _α/2_* = 1.96), power (1 − *β*) = 90% (*Z_β_* = 1.282), baseline VMS incidence before MHT intervention 50–60% (2), and expected 75% reduction in VMS incidence after MHT intervention (16). The calculated minimum sample size was approximately 35 cases; after accounting for a potential 20% loss to follow-up, the final required sample size was adjusted to 42 cases. The actual sample size enrolled in this study was significantly larger than the calculated minimum requirement, which ensured adequate statistical power to detect the 75% reduction in VMS incidence after MHT intervention.

## 3 Results

**Figure 1:**
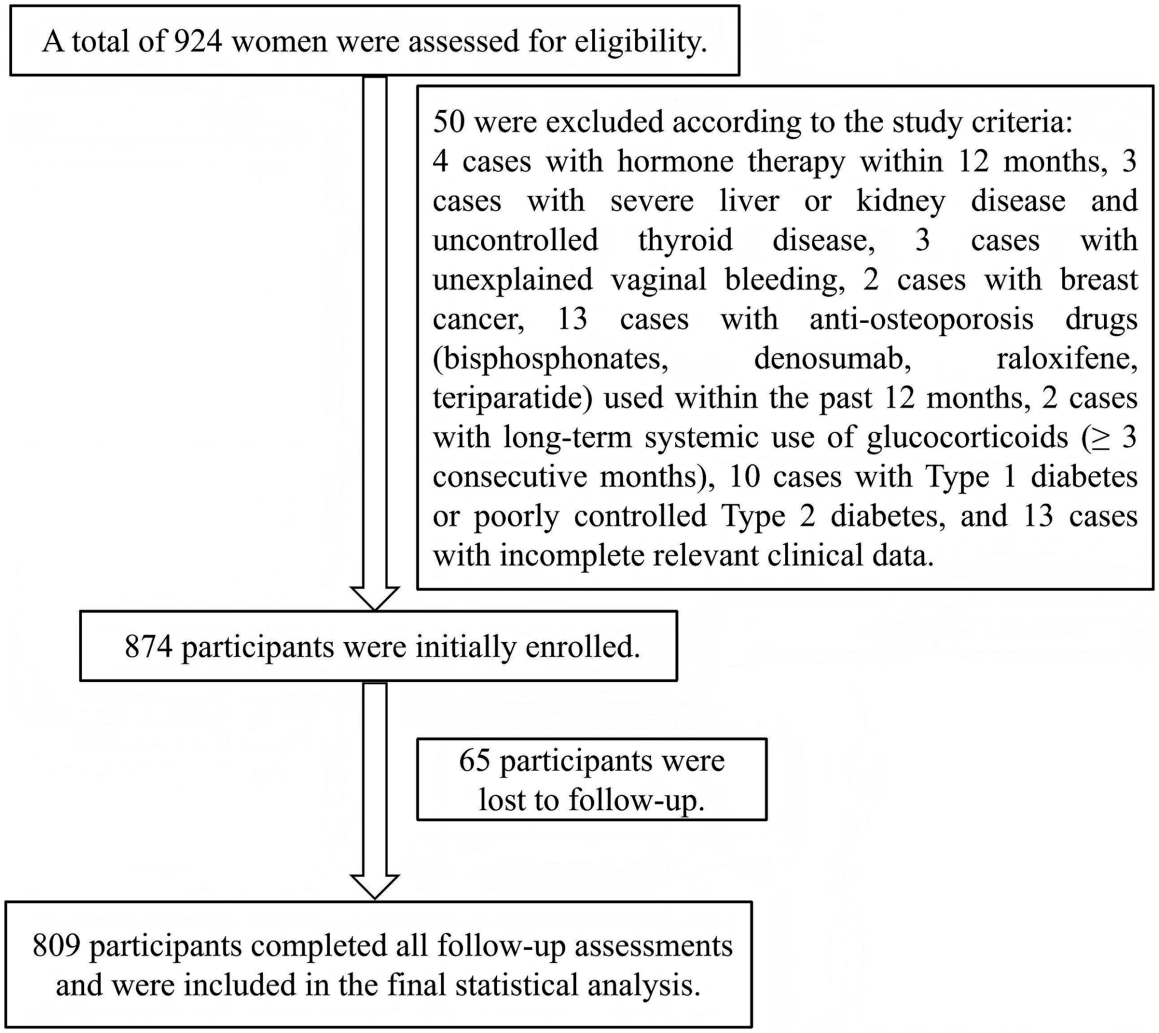
A complete study flow chart.

A total of 924 women were assessed for eligibility. Of these, 50 were excluded according to the study criteria, and 874 participants were initially enrolled. During the 24-month follow-up period, 65 participants were lost to follow-up, and 809 participants completed all follow-up assessments and were included in the final statistical analysis. The detailed study flow chart was summarized in Figure 1.

### 3.1 Characteristics of study population

A total of 809 postmenopausal women were enrolled in this study. Baseline data, including demographic characteristics, menopausal symptoms, and bone mineral density, were collected. The participants had a mean age of 49.78 ± 2.05 years, and the time since menopause was 2.61 ± 1.80 years. In terms of general demographics, 71.0% (*n* = 574) resided in cities, 56.1% (*n* = 454) held a college degree or above, 41.2% (*n* = 333) were employed by government agencies or enterprises/institutions, and 59.2% (*n* = 479) reported a monthly household income exceeding 8,000 yuan. The detailed demographic characteristics were summarized in Table 1.

**Table 1:** Basic information of the research subjects.

Variable	Mean ± SD/*n* (%)
Age, years old	49.78 ± 2.05
Time since menopause (years)	2.61 ± 1.80
Place of residence	
City	574 (71.0%)
Countryside	235 (29.0%)
Education levels	
Below primary school	53 (6.5%)
Junior high school/high school	302 (37.4%)
College degree or above	454 (56.1%)
Occupation status	
Employees of government agencies/enterprises or institutions	333 (41.2%)
Workers or farmers	12 (1.5%)
Business/service practitioners	16 (2.0%)
Freelance/Self-employed	259 (32.0%)
Non-employed individuals (unemployed, retired, full-time housewives)	189 (23.3%)
Monthly household income (CNY)	
<5,000	117 (14.5%)
5,000–8,000	213 (26.3%)
≥8,000	479 (59.2%)
Smoking status	
No	769 (99.5%)
Yes	40 (0.5%)
Serum estradiol (pg/mL)	14.6 ± 5.2
FSH (IU/L)	58.3 ± 12.6
25-hydroxyvitamin D (ng/mL)	21.6 ± 6.8
Fasting glucose (mmol/L)	5.3 ± 0.6

### 3.2 Changes in bone density before and after hormone replacement therapy

At baseline, the mean lumbar spine BMD was 0.912 ± 0.116 g/cm^2^ with a T-score of −0.8 ± 1.1, the mean femoral neck BMD was 0.786 ± 0.104 g/cm^2^ with a T-score of −1.1 ± 1.2, and the mean total hip BMD was 0.864 ± 0.110 g/cm^2^ with a T-score of −0.7 ± 1.1. According to WHO diagnostic criteria, the prevalence of osteoporosis at baseline was 18.0%, and the prevalence of osteopenia was 27.4%. After MHT, the prevalence of osteoporosis at 6, 12, 18, and 24 months was 17.3, 15.7, 14.8, and 14.0%, respectively; the prevalence of osteopenia was 27.0, 24.5, 23.5, and 21.4%, respectively. Friedman M test demonstrated significant differences in bone density grade distribution across the five time points (*M* = 14561.98, df = 4, *p* < 0.001). Details are presented in Table 2.

**Table 2:** Distribution and prevalence of bone density grading at different time points.

Follow-up time	*n*	Normal	Osteopenia	Osteoporosis
Baseline	809	442 (54.6)	221 (27.4)	146 (18.0)
6 months	809	451 (55.7)	218 (27.0)	140 (17.3)
12 months	809	484 (59.8)	198 (24.5)	127 (15.7)
18 months	809	499 (61.7)	190 (23.5)	120 (14.8)
24 months	809	523 (64.6)	173 (21.4)	113 (14.0)

Paired comparisons of bone mineral density were performed between baseline and each post-treatment time point (6, 12, 18, and 24 months). A Bonferroni correction was applied, resulting in an adjusted significance level of *α’* = 0.0125. The two-sided *p*-values for all four comparisons were below this adjusted threshold (*p* < 0.0125), indicating statistically significant differences at each time point. Detailed results are summarized in Table 3.

**Table 3:** Wilcoxon test results for bone density levels before treatment and at various time points after treatment.

Time points	*Z* value	*p* value	Adjusted *p*-value (*α*’ = 0.0125)
Baseline VS 6 months	−3.21	0.0013	<0.0125
Baseline VS 12 months	−6.85	<0.001	<0.0125
Baseline VS 18 months	−8.22	<0.001	<0.0125
Baseline VS 24 months	−10.56	<0.001	<0.0125

After Bonferroni correction, the significance level *α*’ is 0.05/4 = 0.0125.

Comparisons of bone mineral density across post-treatment time points revealed specific pairwise differences. *Post hoc* analyses indicated statistically significant increased between 6 months and each later time point (12, 18, and 24 months), as well as between 12 and 24 months (all *p* < 0.0083). In contrast, no significant differences were found between the 12- and 18-month intervals or between the 18- and 24-month intervals (both *p* > 0.0083). These detailed results are presented in Table 4.

**Table 4:** Pairwise comparison results of bone mineral density grading distribution at different time points (Bonferroni correction).

Time points	*Z* value	*p* value	Adjusted *p*-value (*α*’ = 0.0125)
6 months VS 12 months	−2.86	0.0043	<0.0083
6 months VS 18 months	−4.15	<0.001	<0.0083
6 months VS 24 months	−6.32	<0.001	<0.0083
12 months VS 24 months	−2.11	0.0348	>0.0083
12 months VS 24 months	−3.68	<0.001	<0.0083
18 months VS 24 months	−1.85	0.0642	>0.0083

After Bonferroni correction, the significance level *α*’ is 0.05/6 ≈ 0.0083.

### 3.3 Changes in Kuppermann scores before and after hormone replacement therapy

At enrollment, the mean Kuppermann score for postmenopausal women in this study was 40.17 ± 6.04. The results of repeated measures analysis of variance indicated that there was a significant overall difference in Kuppermann scores at different time points (*F* = 586.324, *df* = 4, *p* < 0.001) (Table 5).

**Table 5:** Changes in Kuppermann scores at different time points.

Follow-up time	*n*	Kuppermann score	Difference from before treatment
Baseline	809	40.17 ± 6.04	–
6 months	809	26.90 ± 6.02	−13.27
12 months	809	22.67 ± 5.72	−17.50
18 months	809	14.54 ± 4.89	−25.63
24 months	809	11.38 ± 4.90	−28.79

The pairwise comparisons of Kuppermann scores across time points were detailed in Table 6. Compared to baseline, Kuppermann scores showed a significant decrease at all post-treatment assessments (6, 12, 18, and 24 months; all *p* < 0.001). While no significant difference was found between the 6- and 12-month assessments (adjusted *p* > 0.005). Significant reductions in scores were observed between 12 and 18 months and between 18 and 24 months (both adjusted *p* < 0.005). Notably, the magnitude of decrease from 18 to 24 months (3.16 points) was smaller than that from 12 to 18 months (8.13 points).

**Table 6:** Pairwise comparison results of Kuppermann scores at different time points (Bonferroni correction).

Time points	Mean difference (95%*CI*)	*T* value	*p* value	Adjusted *p-*value
Baseline VS 6 months	13.27 (12.35–14.19)	28.642	<0.001	<0.001
Baseline VS 12 months	17.50 (16.58–18.42)	37.895	<0.001	<0.001
Baseline VS 18 months	25.63 (24.71–26.55)	55.421	<0.001	<0.001
Baseline VS 24 months	28.79 (27.87–29.71)	62.153	<0.001	<0.001
6 months VS 12 months	4.23 (3.31–5.15)	9.253	0.002	>0.005
6 months VS 18 months	12.36 (11.44–13.28)	26.779	<0.001	<0.001
6 months VS 24 months	15.52 (14.60–16.44)	33.511	<0.001	<0.001
12 months VS 18 months	8.13 (7.21–9.05)	17.526	<0.001	<0.005
12 months VS 24 months	11.29 (10.37–12.21)	24.258	<0.001	<0.005
18 months VS 24 months	3.16 (2.24–4.08)	6.732	<0.001	<0.005

A higher Kuppermann score indicates more severe clinical symptoms; the significance level after Bonferroni correction is *α* = 0.05/10 = 0.005.

### 3.4 Changes in breast density, breast fibrous tissue volume, and breast volume before and after hormone replacement therapy

After hormone replacement therapy, follow-up visits were conducted at 6 months, 12 months, 18 months, and 24 months. There were no statistically significant differences in breast density, breast fibrous tissue volume, and breast volume as analyzed by repeated measures analysis (all *p* values ≥ 0.05), as detailed in Table 7.

**Table 7:** Changes in the breasts before and after hormone replacement therapy.

Breast composition parameters	Baseline	6 months	12 months	18 months	24 months
Breast density (%)	17.23 ± 6.92	19.73 ± 6.69	17.46 ± 6.63	19.20 ± 8.52	19.87 ± 7.19
Breast fibrous tissue volume (cm^3^)	145.08 ± 76.00	158.23 ± 86.50	160.29 ± 84.59	149.08 ± 83.44	150.58 ± 70.37
Breast volume (cm^3^)	872.50 ± 442.57	861.60 ± 425.87	851.27 ± 382.20	873.69 ± 438.01	869.50 ± 442.57

After hormone replacement therapy, the prevalence of breast calcification and breast masses showed no statistical difference at 6, 12, 18, and 24 months of follow-up, as determined by Cochran’s *Q* test (all *p* values ≥ 0.05), as detailed in Table 8.

**Table 8:** Changes in breast calcification and mass before and after hormone replacement therapy.

Breast imaging findings	Baseline	6 months	12 months	18 months	24 months
Breast calcification	150 (18.6%)	156 (19.3%)	160 (19.8%)	162 (20.0%)	165 (20.4%)
Breast lump	315 (38.9%)	316 (39.1%)	318 (39.3%)	317 (39.2%)	320 (39.6%)

## 4 Discussion

A total of 809 participants were enrolled in this study, with a mean age of (49.78 ± 2.05) years. This result was consistent with the average menopausal age of (48.7 ± 4.3) years reported in a cross-sectional study involving 300,000 Chinese women (17), which validated the rationality of the age distribution of the study subjects. It was also comparable to the average menopausal age documented in international studies (18, 19), laying a solid age-matched foundation for subsequent efficacy analyses.

In terms of demographic characteristics, 71% of the participants resided in urban areas, 56.1% held a college degree or above, 41.2% were employed by government agencies or enterprises, and 59.2% had a monthly household income of ≥8,000 yuan. These data indicated that urban populations with higher educational attainment and household income generally exhibited stronger health awareness, paid greater attention to menopausal symptoms, and demonstrated a higher willingness to seek medical intervention, thus constituting the primary group receiving MHT in China currently. Significantly, women make up half of the workforce, and about 80% of menopausal women experience problematic symptoms whether they are in the workforce or not (20, 21), which highlights the importance of MHT intervention in alleviating menopausal distress and maintaining women’s quality of life and work ability.

Bone health protection is regarded as one of the core benefits of MHT. Notably, the baseline incidence rates of osteoporosis and osteopenia in this study were 18.0 and 27.4%, respectively. Although the incidence of osteoporosis was slightly lower than the national epidemiological survey data, which reported that approximately 20.7% of postmenopausal women over 50 years of age suffered from osteoporosis (22), the total incidence of bone mass abnormalities (including osteoporosis and osteopenia) reached 45.4%. This finding suggested that the study population was already facing a significant risk of bone health impairments, which was notably higher than that of women over 50 years of age in North America (23).

Follow-up data revealed that bone mineral density (BMD) showed a statistically significant increase at 12 months after the initiation of MHT, and the improvement effect continued to enhance with the extension of treatment duration. The proportion of participants with normal BMD increased from 64.6 to 70.7% at 24 months of treatment, demonstrating a time-dependent pattern of BMD improvement via MHT.

The decline in ovarian function in perimenopausal women leads to a sharp decrease in estrogen levels, which is the core trigger for bone loss. Estrogen binds to estrogen receptors on the surface of osteoblasts and osteoclasts, thereby inhibiting the differentiation and activity of osteoclasts and reducing bone resorption. Meanwhile, it promotes the secretion of osteoprotegerin by osteoblasts, further blocking the bone resorption signaling pathway (24). Evidence-based medical evidence has confirmed that MHT exerts a protective effect on bone health (9, 25, 26). By inhibiting osteoclast activity and reducing bone turnover, MHT prevents rapid postmenopausal bone loss and osteoporosis, and lowers the risk of fractures.

The results of this study showed that BMD continued to increase with the prolongation of MHT after 1 year of intervention. Essentially, this may be attributed to the fact that MHT re-establishes the balance between bone resorption and bone formation through exogenous estrogen supplementation, and this balance tends to shift more significantly toward bone formation dominance as the treatment cycle extends. In addition, the findings of this study also suggested that for patients with baseline osteopenia, clinicians should recommend completing at least 12 months of hormone therapy to achieve a significant improvement in BMD.

The modified Kuppermann score is a classic tool for assessing the severity of menopausal symptoms, encompassing 13 core symptoms such as hot flashes, night sweats, insomnia, mood swings, and vaginal dryness. Dynamic changes in its scores directly reflect the symptom-improving efficacy of the treatment regimen (27, 28). In this study, the baseline Kuppermann score of the enrolled participants was (40.17 *±* 6.04) points, indicating a severe symptom level. However, the score decreased significantly to (26.90 *±* 6.02) points at 6 months after MHT, reducing from severe to moderate. The scores continued to decline at 12, 18, and 24 months of treatment, suggesting that the symptom relief effect became more prominent with prolonged treatment, which was highly consistent with the pathological mechanism by which estrogen deficiency induces menopausal symptoms.

Estrogen deficiency during menopause can lead to autonomic nervous system dysfunction, triggering vasomotor symptoms such as hot flashes and night sweats (29). Simultaneously, it affects the synthesis and metabolism of central nervous system neurotransmitters (e.g., serotonin and dopamine), resulting in mental and psychological symptoms including insomnia, anxiety, and depression (30). It can also cause mucosal atrophy of the genitourinary system, leading to complications such as vaginal dryness and dyspareunia (31). Crucially, Menopausal Hormone Therapy is widely recognized as the most effective pharmacological treatment modality to reduce bothersome issues of vasomotor symptoms and cognitive dysfunction in peri-menopausal, menopausal, and post-menopausal women, and its clinical application abides by the state of the art of internationally accepted post-reproductive health guidelines. A large number of clinical studies and evidence-based guidelines have confirmed this conclusion: Schneider and Birkhäuser pointed out in their research on climacteric women’s quality of life that MHT can effectively alleviate various menopausal symptoms, especially vasomotor symptoms, and improve women’s quality of life (32); Dalal and Agarwal also emphasized in their study on postmenopausal syndrome that MHT is the first-choice pharmacological intervention for relieving vasomotor symptoms such as hot flashes and night sweats (33). In addition, Kaunitz and Manson summarized in their research on menopausal symptom management that MHT not only relieves vasomotor symptoms but also has a positive regulatory effect on cognitive function, which can alleviate cognitive dysfunction such as memory loss caused by estrogen deficiency (5). Birkhäuser and Haenggi further confirmed the benefits of different routes of MHT administration, all of which can effectively reduce menopausal-related distresses when used in accordance with clinical guidelines (34). More importantly, the Endocrine Society’s scientific statement on postmenopausal hormone therapy clearly recommends MHT as the most effective pharmacological method for relieving vasomotor symptoms and improving cognitive function in menopausal women, and specifies the standardized application principles to ensure its safety and effectiveness (35). This conclusion is further supported by the results of this study, where MHT significantly reduced Kuppermann scores (from 40.17 ± 6.04 points at baseline to 26.90 ± 6.02 points at 6 months) and effectively alleviated VMS and related cognitive and psychological symptoms, which is consistent with the international consensus on MHT’s efficacy and the recommendations of post-reproductive health guidelines.

Hormone replacement therapy exerts a direct effect on the autonomic nerve center and genitourinary mucosa through exogenous estrogen supplementation, rapidly alleviating vasomotor and genitourinary symptoms (36). Meanwhile, it gradually improves mental and psychological symptoms by regulating neurotransmitter balance (37). The results of this study showed that a significant decrease in Kuppermann scores was observed at 6 months of hormone therapy, which could quickly relieve core distresses of patients such as hot flashes, sweating, insomnia, and anxiety, thereby improving treatment adherence. The scores further decreased and the improvement amplitude expanded at 18–24 months of treatment, indicating that the regulatory effect of MHT on the neuroendocrine system has a cumulative effect, and long-term treatment can achieve in-depth symptom relief.

Combined with the analysis of the demographic characteristics of the study population, 65.1% of the participants had a junior high school education or below. These patients may have insufficient awareness of menopause-related knowledge and are prone to negative emotions such as anxiety and depression when symptoms are severe. Through rapid and sustained symptom improvement, MHT can not only improve patients’ quality of life but also indirectly enhance their mental health status, thus presenting important dual physical and mental benefits.

Breast safety has always been a core concern in the clinical application of MHT, with conflicting viewpoints (38, 39). Some studies have indicated that MHT may increase the risk of breast cancer, while others have drawn the opposite conclusion, suggesting that MHT does not significantly elevate the incidence of breast cancer in women.

This study provided key evidence from two aspects: changes in breast morphology and the risk of breast lesions. On the one hand, no significant changes were observed in breast density, breast fibrous tissue volume, or breast volume at 6–24 months after MHT. On the other hand, the incidence of breast calcification and masses did not show significant fluctuations during the follow-up period. These results were consistent with the viewpoint proposed by Santoro that “standard low-dose MHT is not significantly associated with the risk of breast diseases” (40), and also provided direct cohort study evidence to address safety concerns in the clinical application of MHT.

However, it is necessary to objectively acknowledge that the conclusion on breast safety in this study was based on 24-month follow-up results. Further research is required to verify the breast safety of long-term hormone use exceeding 5 years. Venous thromboembolism was not evaluated as a long-term outcome. Meanwhile, this study did not include high-risk groups with a family history of breast cancer or a past history of benign breast lesions, and the breast-related risks of MHT in such populations remain unclear. Additionally, as cardiovascular safety is a concern in the clinical application of MHT, this study did not conduct relevant monitoring and analysis of cardiovascular indicators. Bone turnover markers (*β*-CTX, PINP, PTH) and renal function indicators (eGFR) were not routinely measured in clinical practice, so these data were not collected. These limitations restrict the comprehensiveness of the safety assessment of MHT in this study. Future studies should be specifically conducted on high-risk groups and incorporate systematic cardiovascular safety monitoring to improve the individualized assessment and treatment system of MHT.

## 5 Conclusion

In conclusion, this 2-year real-world cohort study demonstrates that a standardized 24-month MHT regimen effectively improves bone mineral density, alleviates menopausal symptoms in a distinct stage-dependent manner, and exhibits a favorable breast safety profile within the treatment period. These findings provide robust clinical evidence supporting MHT as a beneficial intervention for the comprehensive management of postmenopausal health in Chinese women, particularly in ameliorating climacteric symptoms and preventing bone loss.

## Data Availability

The datasets presented in this article are not readily available because the dataset contains private and sensitive information of research participants, and its disclosure is prohibited by ethics committee regulations and institutional policies. Therefore, no access request can be processed.
